# Supplementary material for: Generation of a Normal Long-Term-Cultured Chinese Hook Snout Carp Spermatogonial Stem Cell Line Capable of Sperm Production In Vitro
Source: Biology (Basel). 2022 Jul 18;11(7):1069. doi: 10.3390/biology11071069 (PMC9312933; doi:10.3390/biology11071069)
Supplement: Supplementary file 1 [file biology-11-01069-s001.zip › biology-1806858-supplementary.pdf]

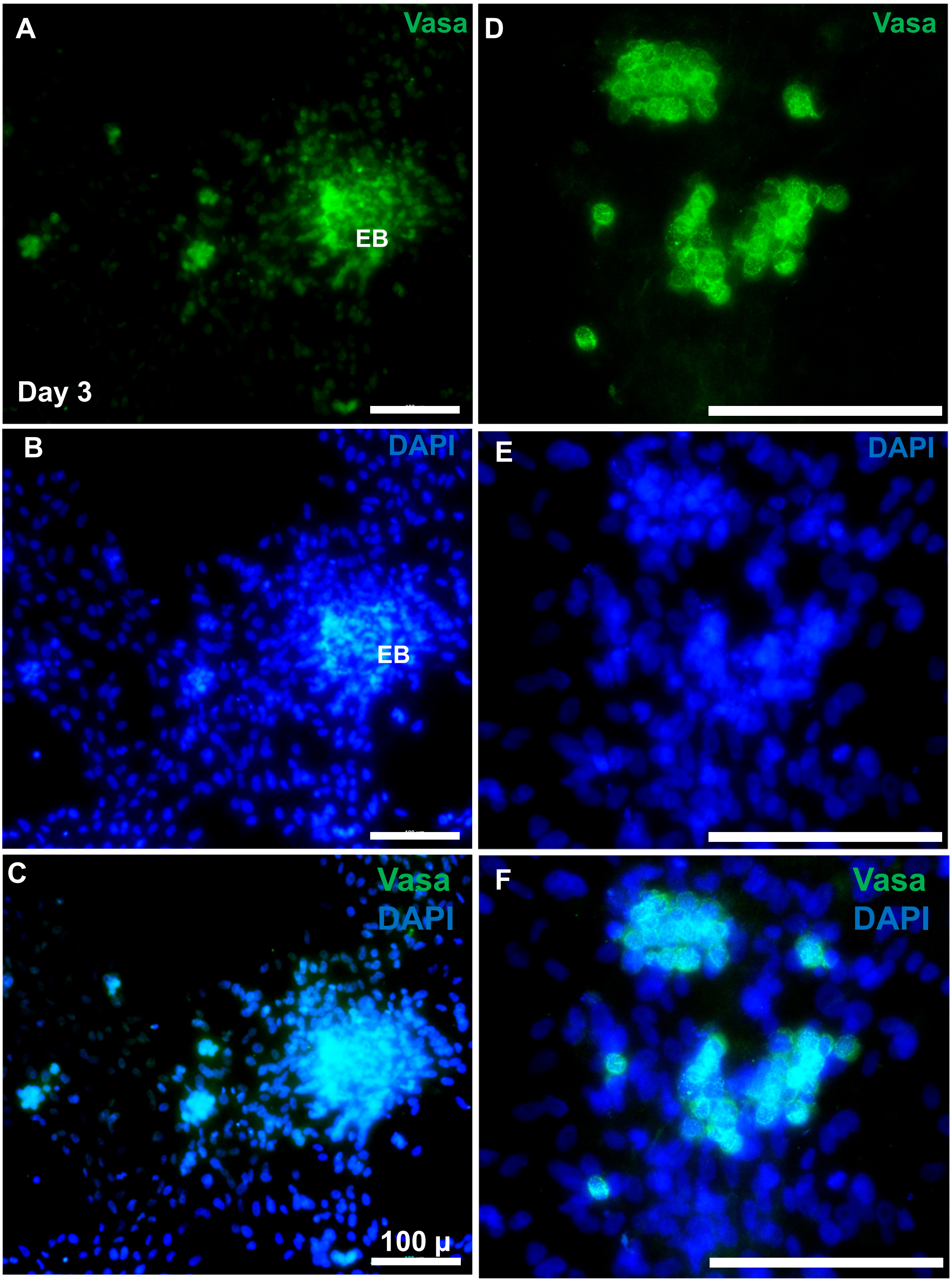

Supplementary Figure S1. Immunofluorescence analysis of primary cultured testis cells *of O. bidens*. Green fluorescence is for Vasa protein and blue is the nuclear staining with DAPI. (A-C) View under the low magnification. (D-F) View under the high magnification. EB, Embryoid body. Scale bars = 100 µm.

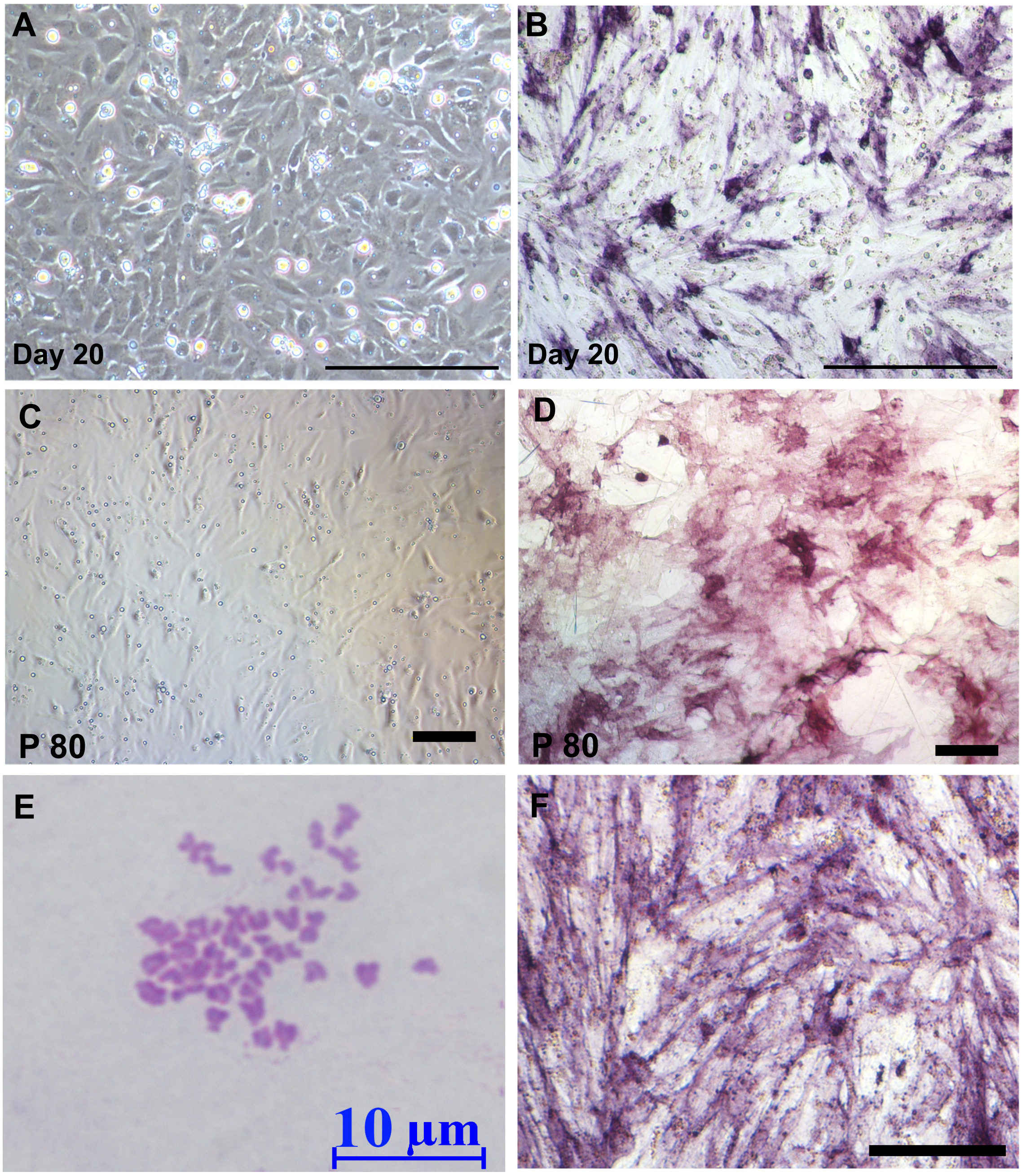

Supplementary Figure S2. Derivation and characterization of ObSSCs. (A-B) Alkaline phosphatase staining analysis of ObSSC cells at passage 6 (Day 20). (C-D) ObSSCs were recovered and stained by alkaline phosphatase after cryopreservation. (E) Haploid metaphase of ObSSCs. (F) AP staining analysis of RFP-expressing ObSSCs. Scale bars = 100 µm.

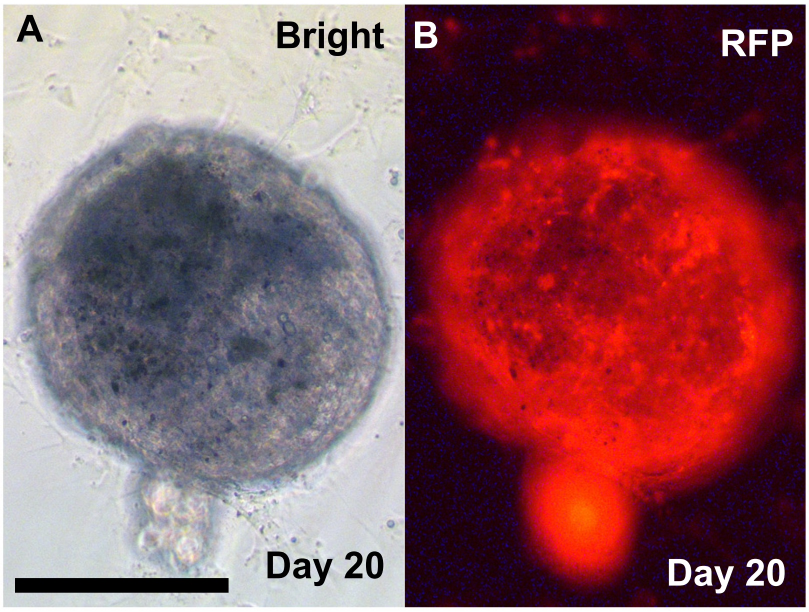

Supplementary Figure S3. Gonadal-like organoids formed after co-culture of ObSSCs and ObTCs for 20 days. (A) Bright field. (B) Fluorescence field. Scale bars = 100 μm.

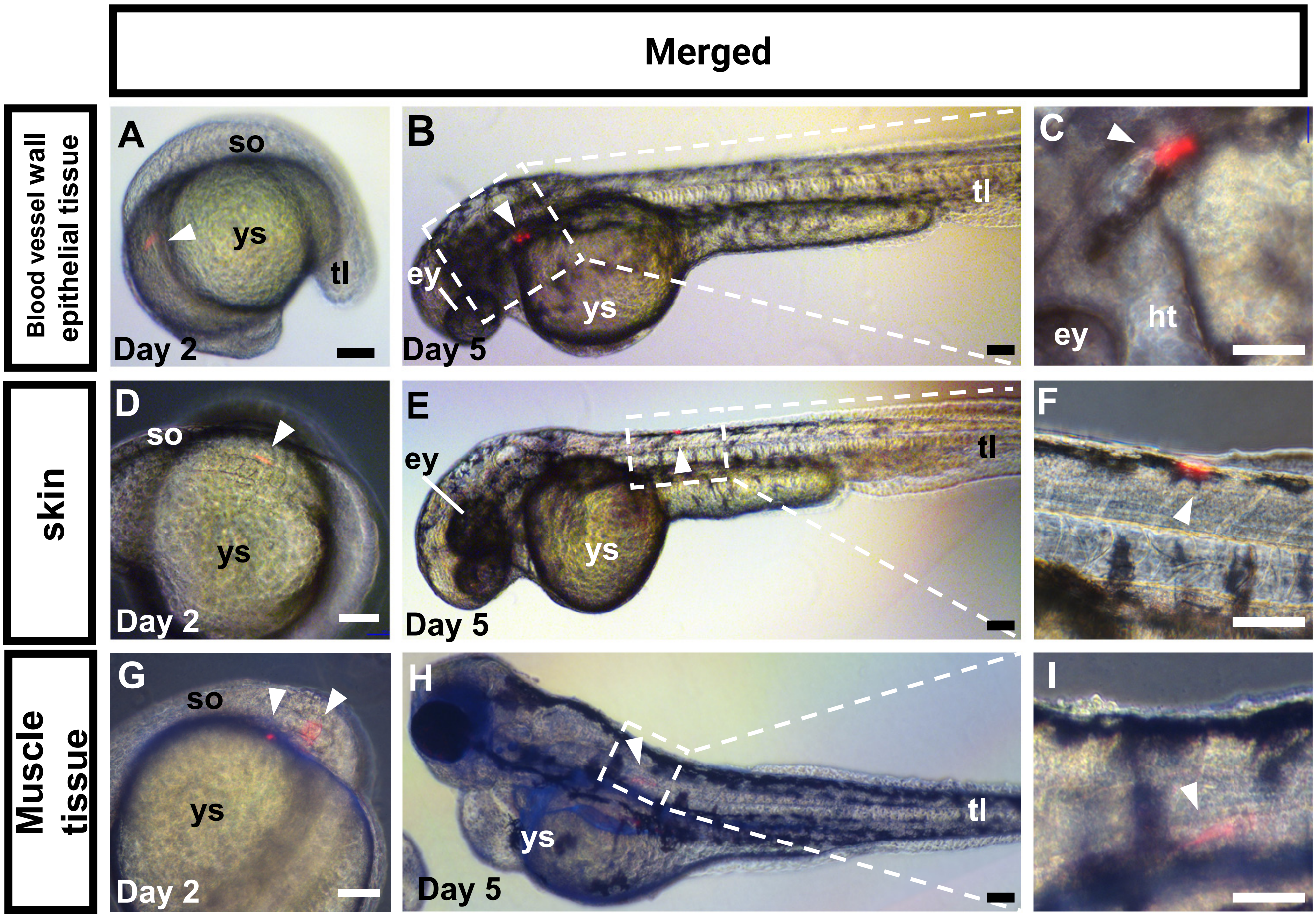

Supplementary Figure S4. Pluripotency of ObSSCs *in vivo*. The RFP-expressing ObSSCs were transplanted into zebrafish embryos at blastula stage. (A–I) Showing micrograph of a zebrafish chimera in the side view from 2 dpf to 5 dpf. (A–C, D-F and G-I) Three representative chimeras at different developmental days. Showing wide distribution of ObSSCs in zebrafish embryos including blood vessel wall epithelial tissue, skin, muscle tissue etc. The photomicrograph is a merger of red fluorescence and bright fields. ey, eye; ht, heart; so somite; tl, tail; ys, yolk sac. Scale bars = 100 μm.
